# Colorado State Dental Association

**Published:** 1893-08

**Authors:** P. T. Smith

**Affiliations:** Denver


### COLORADO STATE DENTAL ASSOCIATION.

The Colorado State Dental Association held its Eighth Annual Meeting in Denver, commencing on June 6, and remained in session until the afternoon of the 9th, during which time eleven very well-prepared and interesting papers were read, and many elicited animated discussion.

The construction of bridges was exemplified in the clinics in a creditable manner.

Dr. S. Davis, of Denver, exhibited a new and novel dowel crown, with platina band, which formed at once a cap and band neatly encompassing the end of a prepared root. After adjustment with the dowel in socket and band in place, a common plate tooth was carefully arranged and secured to band and dowel by means of wax, when the whole structure was removed and invested. After crystallization the wax was removed, and a low flowing grade of body was easily melted over the palatal surface of the tooth, incorporating the pins in the tooth, the end of the dowel passing down by the pins, the platina cap, and the band.

After cooling, the investment was removed and the band covered with gum-colored body and beautifully flowed, so that when the combined tooth, band, and dowel was adjusted on the root no defect in the gingival border could be easily detected. The structure seemed as strong and more desirable than the Logan crown, as it possesses the advantage of the band covered by gum-colored enamel in such an artistic manner as to dissemble its artificial character.

An election of officers for the ensuing year resulted in the selection of P. T. Smith, Denver, President; W. S. Brennaman,

Leadville, First Vice-President; W. R. Sinton, Colorado Springs, Second Vice-President; A. H. Sawins, Denver, Recording Secretary; Mrs. S. M. Townsend, Denver, Corresponding Secretary; William Smedley, Denver, Treasurer. M. A. Bartleson, Denver, was elected delegate to World's Columbian Dental Congress. Glenwood Springs, a most delightful mountain summer resort, was selected for the next annual meeting in June, where it is hoped the American Dental Association can be induced to hold their annual session of 1894. A more favorable place could not be found in all the accessible parts of America.

P. T. Smith.

Denver.

